# Activation of Nuclear Factor-κB in the Brain after Experimental Subarachnoid Hemorrhage and Its Potential Role in Delayed Brain Injury

**DOI:** 10.1371/journal.pone.0060290

**Published:** 2013-03-25

**Authors:** Wan-Chun You, Chun-xi Wang, Yun-xi Pan, Xin Zhang, Xiao-ming Zhou, Xiang-sheng Zhang, Ji-xin Shi, Meng-liang Zhou

**Affiliations:** Department of Neurosurgery, Jinling Hospital, School of Medicine, Nanjing University, Nanjing, Jiangsu Province, China; Charité-Universitätsmedizin Berlin, Germany

## Abstract

It has been reported that inflammation is involved in brain injury after subarachnoid hemorrhage (SAH). Nuclear factor-κB (NF-κB) is a key transcriptional regulator of inflammatory genes. Here, we used pyrrolidine dithiocarbamate(PDTC), an inhibitor of NF-κB, through intracisternal injection to study the role of NF-κB in delayed brain injury after SAH. A total of 55 rabbits were randomly divided into five groups: the control group; the SAH groups including Day-3, 5, and 7 SAH groups (the rabbits in these groups were sacrificed at 3, 5, 7 days after SAH, respectively); and the PDTC group (n = 11 for each group). Electrophoretic mobility shift assay (EMSA) was performed to detect NF-κB DNA-binding activity. The mRNA levels of tumor necrosis factor (TNF)-α, interleukin (IL)-1β, and intercellular adhesion molecule (ICAM)-1 were evaluated by RT-PCR analysis. Deoxyribonucleic acid fragmentation was detected by TUNEL and p65 immunoactivity was assessed by immunohistochemistry. Our results showed the activation of NF-κB after SAH, especially at day 3 and 5. The activated p65 was detected in neurons. NF-κB DNA-binding activity was suppressed by intracisternal administration of PDTC. Increased levels of the TNF-α, IL-1β, and ICAM-1 mRNA were found in the brain at day 5 after SAH, and which were suppressed in the PDTC group. The number of TUNEL-positive cells also decreased significantly in the PDTC group compared with that in the Day-5 SAH group. These results demonstrated that the activated NF-κB in neurons after SAH plays an important role in regulating the expressions of inflammatory genes in the brain, and ultimately contributes to delayed brain injury.

## Introduction

In most developed countries, stroke is the third leading cause of death, 20% of which are due to aneurysmal subarachnoid hemorrhage (SAH)[1]. There are far more studies focusing on the contraction of the cerebral arteries than on the pathophysiological changes in the brain after SAH, which also could lead to disastrous outcomes.

Early and delayed brain injury after SAH have been well documented, but the underlying mechanisms still remain unclear. It has been reported that cell death, especially apoptosis, occurs in the brain after exposure to subarachnoid hemolysate or hemorrhage. A previous study demonstrated that SAH could trigger an inflammatory reaction in the brain, which might contribute to cell death[2]. Additionally, increasing evidence indicates that inflammatory cytokines and adhesion molecules, such as tumor necrosis factor (TNF)-α, interleukin (IL)-1, IL-6, IL-8, IL-10, intercellular adhesion molecule (ICAM)-1, and vascular cell adhesion molecule (VCAM)-1, are increased in the cerebrospinal fluid (CSF) after SAH[3]. These increased cytokines and adhesion molecules in CSF are correlated with neurological injury, so it is likely that they contribute to brain injury after SAH[4], [5].

Transcription factor nuclear factor-κB (NF-κB) plays a crucial role in regulating the immunity and inflammation in the central nervous system (CNS)[6]. NF-κB has been reported to be involved in traumatic brain trauma[7], spinal cord injury[8], and ischemia[9], which all share many common pathological events with early and delayed brain injury after SAH. Thus, it seems likely that NF-κB may contribute to brain injury after SAH as well.

Among all chemicals or drugs which could inhibit NF-κB, including glucocorticoids[10], [11], vitamin C [12], vitamin E[13], flavonoids [14], leflunomide [15], cyclosporin A and tacrolimus (FK-506) [16], pyrrolidine dithiocarbamate (PDTC) [17] has been used more widely in the experimental study as the inhibitor of NF-κB for the treatment of inflammatory diseases [18]. Moreover, it has been reported that PDTC could protect against brain ischemia with a wide therapeutic time window via inhibiting the activation of NF-κB in neurons [19]. The κB site decoy oligodeoxynucleotides (ODNs), which blocks the binding of NF-kB to DNA, are also used in a number of animal studies[20], [21]. However, ODNs are degraded rapidly *in vivo*, therefore high doses of ODNs have to be delivered frequently which prevents their clinical use. NF-κB could be suppressed by means of RNA silencing, but this method need to be evaluated more precisely *in vivo*.

Thus, the present study was designed to assess the change of NF-kB DNA-binding activity in the brain after SAH and then to determine the potential role of NF-kB in delayed brain injury after SAH. In addition, the influence of PDTC on delayed brain injury after SAH was also investigated.

## Materials and Methods

This study was carried out in strict accordance with the recommendations in the Guide for the Care and Use of Laboratory Animals of the National Institutes of Health. The protocol was approved by the Animal Care and Use Committee of Nanjing University. All surgeries were performed under anesthesia, and all efforts were made to minimize suffering. Fifty-five male rabbits were randomly assigned to 5 groups:

The animals in group 1 served as controls and intracisternal saline injection was performed (n = 11).

The animals in groups 2, 3, 4 were subjected to experimental SAH on *day 0* and *2* twice and killed on *days 3, 5, and 7,* respectively (n = 11 for each group).

The animals in group 5 were subjected to experimental SAH on *day 0* and *2* twice, intracisternal administration of PDTC (3 mg/kg) from *day 0* to *day 5*, and then sacrificed on *day 5* (n = 11).

Five rabbits in each group were killed for detection the localization of activated NF-κB by immunohistochemical assay of p65 and for detection of deoxyribonucleic acid (DNA) fragmentation by terminal deoxynucleotidyl transferase-mediated deoxyuridine triphosphate nick end labeling (TUNEL). Three rabbits were sacrificed for detection of NF-κB DNA-binding activity by electrophoretic mobility shift assay (EMSA). Others were used for detecting the mRNA of TNF-α, IL-1β, and ICAM-1 by semi-quantitative reverse transcriptase polymerase chain reaction (RT-PCR) analysis.

### Induction of Experimental SAH

In groups 2, 3, 4, and 5, SAH was produced according to the two-hemorrhage method [22]. The rabbits were anaesthetized with an intramuscular injection of a mixture of ketamine (25 mg/kg) and droperidol (1.0 mg/kg) on *day 0*. Under spontaneous breathing, a 21-gauge butterfly needle was percutaneously inserted into the cisterna magna. After withdrawal of 1.5 ml of the CSF, the same amount of non-heparinized fresh autologous auricular arterial blood was injected into the cisterna magna by a 1-min slow injection under aseptic technique. The animals were then kept in a 30° head-down position for 30 min. Once being recovered from anesthesia, they were returned to the feeding room. Forty-eight hours after the first SAH, the second injection was accomplished using the same procedure as described above.

In the animals of the control group, the same technique was applied with injection of sterile saline instead of blood. In the PDTC group, PDTC (3 mg/kg) was injected into cisterna magna in the blood injection manner every 24 hours from 1 min before first blood injection on *day 0* to *day 5*.

On *days 3, 5* and *7*, the rabbits scheduled for sacrifice were anesthetized again with an intramuscular injection of a mixture of ketamine (40 mg/kg) and droperidol (2.5 mg/kg). The rabbits were exsanguinated and decollated. The brain was removed and certain areas were taken for assay. The tissue for EMSA and RT-PCR was frozen in liquid nitrogen immediately, while the tissue for TUNEL and immunohistochemistry was fixed with 10% neutral buffered formalin.

### Nuclear protein extracts and EMSA

Nuclear protein of the tissue was extracted and quantified as described [7]. EMSA was performed using a commercial kit (Gel Shift Assay System; Promega, Madison, WI). Consensus oligonucleotide probe (5′-AGT TGA GGG GAC TTT CCC AGG C-3′) was end-labeled with T4-polynucleotide kinase. Nuclear protein (30 µg) was preincubated in a total volume of 9 µl in a binding buffer for 10 min at room temperature. After adding of the ^32^P-labled oligonucleotide probe, the incubation was continued for 20 min. Reaction was stopped by adding 1 µl of gel loading buffer and the mixture was subjected to nondenaturing 4% polyacrylamide gel electrophoresis in 0.5×TBE(Tris-borate-EDTA) buffer. After electrophoresis was conducted at 390 V for 1 h, the gel was vacuum-dried and exposed to X-ray film (Fuji Hyperfilm) at −70°C.

### Immunohistochemical staining

The brain tissue was fixed with the 10% neutral buffered formalin and embedded in paraffin. The tissue sections were used for immunohistochemical staining, which was performed with an anti-p65 polyclonal antibody (diluted 1⊗200, Santa Cruz Biotechnology, Santa Cruz, CA). The sections were incubated with the diluted antibody overnight at 4°C, and then washed, and blocked with 1.6% H_2_O_2_ in phosphate-buffered saline (PBS) for 10 min. After washing with PBS, each section was incubated with horseradish peroxidase (HRP)-conjugated IgG (diluted 1⊗500, Santa Cruz Biotechnology, Santa Cruz, CA) for 60 min at room temperature. Diaminobenzidine (DAB) was used as chromogen and counterstaining was done with hematoxylin.

### RNA extraction and RT-PCR

The levels of TNF-α, IL-1β, and ICAM-1 mRNA expression were determined by RT-PCR. Total RNA was extracted with TriPure Reagent (Roche Diagnostics Corp., Indianapolis, IN, USA) according to the manufacture's instruments. The cDNA synthesis from the isolated RNA was performed using a reverse transcriptional system. Briefly, 4 µg of total RNA was subjected to the first strand cDNA synthesis for 1 h at 42°C in a 20 µl reaction mixture containing 20 U RNase inhibitor, 0.04 µmol of each dNTP, 0.5 µg oligo(dT)_15_ and 15 U AMV reverse transcriptase (Promega). The reaction was terminated by incubation at 95°C for 5 min. The cDNA was stored at –20°C or used for PCR immediately. PCR amplification was performed in a total volume of 25 µl containing 4 µl cDNA, 0.05 µmol MgCl_2_, 2.5 U Taq polymerase, 0.02 µmol of each dNTP, specific oligonucleotide primers(Table 1) for TNF-α, IL-1β, ICAM-1 or GAPDH genes, and 2.5 µl 10×Taq polymerase reaction buffer. Each PCR cycle included a denaturation step at 94°C for 30 s, a primer annealing step for 30 s (the temperatures were listed on Table 1), an extension step at 72°C for 50 s, and a final extension step at 72 °C for 7 min. The number of cycles was also shown in the Table 1. Thereafter, the amplified fragments were detected by agarose gel electrophoresis and visualized by ethidium bromide staining. The gel was captured as a digital image and analyzed using Scion Image software (Scion Corp, Maryland, USA). Values for each sample were normalized by the GAPDH as the control.

**Table 1:** Sequences of PCR primers.

Target gene	Sense primer (5′ to 3′)	Antisense primer (5′ to 3′)	Annealing temperature (°C)	Number of cycles	Size (bp)
TNF-α	GCTGCACTTCAGGCTGATC	CTTGTTCGGGTAGGAGACG	57	34	352
IL-1β	ATCTCCTGCCAACCCTACA	CTTTCAGCTCATA CGTGCC	54	34	274
ICAM-1	GCGGCTCAGTGTCTCATTCC	CACGCAGTCCTCGGCTTCT	58	34	184
GAPDH	GGAGCCAAAAGGGTCATC	CCAGTGAGTTTCCCGTTC	57	30	347

### Assessment of brain injury

The DNA fragmentation of the brain was detected by TUNEL. The coronal sections were embedded in paraffin and sectioned at 4- µm thickness. In situ cell death detection Kit POD (ISCDD, Boehringer Mannheim, Germany) was used according to the manufacturer's instruction. Briefly, paraffin-embedded sections were mounted on positively charged slides, deparaffinized, rehydrated, and washed thoroughly with distilled water. The tissues were digested with 20 g/ml proteinase K (Boehringer Mannheim, Mannheim, Germany) at room temperature for 15 min to retrieve antigen. Endogenous peroxidase activity was blocked by incubation in 0.3% hydrogen peroxide/methanol in phosphate-buffered saline at 37°C for 30 min. The sections were then incubated with terminal deoxynucleotidyl transferase at 37°C for 60 min, which would add the dioxigenin-conjugated dUTP to the 3′-OH ends of fragmented DNA. Anti-dioxigenin antibody peroxidase was applied to the sections to detect the labeled nucleotides. The sections were stained with DAB and counterstained slightly with hematoxylin. The positive cells were identified, counted, and analyzed under a light microscope by another investigator in a blinded fashion. The extent of brain injury was evaluated by calculating the average positive neuronal cells per 100 neurons, and at least 1000 neurons were counted.

### Statistical analysis

All data were presented as mean ± SEM. Software SPSS 11.0 was used for the statistical analysis. Comparison between different groups was made by one-way analysis of variance (ANOVA), followed by Tukey's multiple comparisons test if a significant difference had been determined by ANOVA. A probability value of P<0.05 was considered statistically significant.

## Results

### Time course of NF-κB DNA-binding activity in the brain after SAH

**Figure 1:**
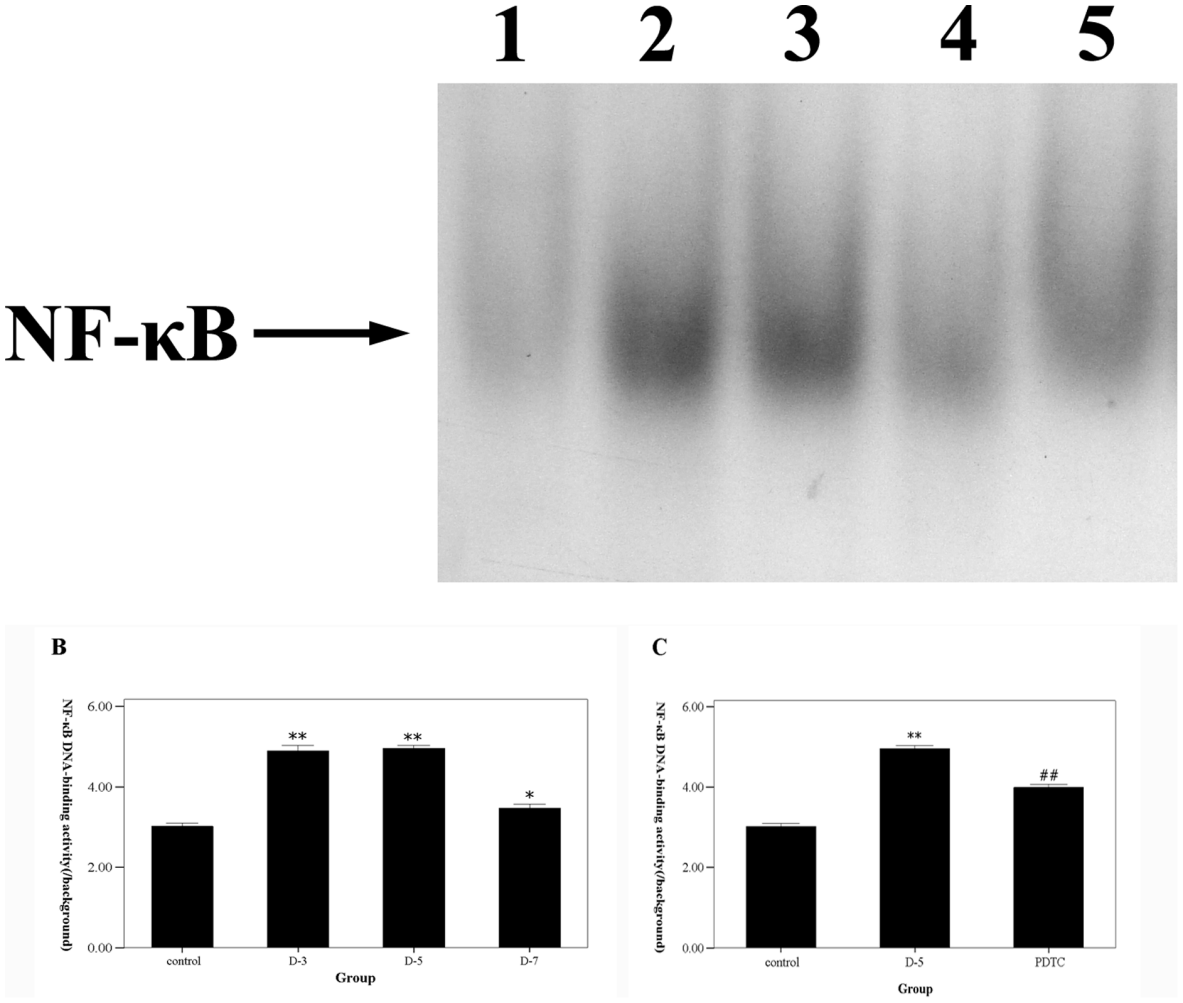
The time course of NF-κB DNA-binding activity detected by EMSA after SAH and the effects of PDTC on NF-κB activation. A. The representative autoradiogram showed the NF-κB DNA-binding activity in each group. B. The time course of NF-κB DNA-binding activity after SAH. Quantification of the DNA-binding activity of NF-κB was performed by densitometric analysis. The NF-κB DNA-binding activity was up-regulated significantly after SAH, especially on day 3 and 5, while restored on day 7. C. the effects of intracisternal administration of PDTC on NF-κB DNA-binding activity. It was shown that NF-κB DNA-binding activity was suppressed after treatment with PDTC. 1 stands for the control group. 2, 3 and 4 stand for the D-3, 5 and 7 SAH groups. 5 stands for the PDTC groups. Results are represented as means ± SEM from five independent experiments in each group. *P<0.05 vs. control group, **P<0.01 vs. control group, #P<0.05 vs. D-5 SAH group.

The temporal activation NF-κB in the brain after SAH was examined by EMSA. The NF-κB DNA-binding activity was up-regulated significantly after SAH compared with that in the control group (P<0.05 vs. control group), especially on *day 3* and *5* (P<0.01 vs. control group)(Fig.1A and B). The increased NF-κB DNA-binding activity on *day 7* after SAH was not more than that on the *day 3* and *day 5* SAH groups.

### Localization of activated NF-κB

**Figure 2:**
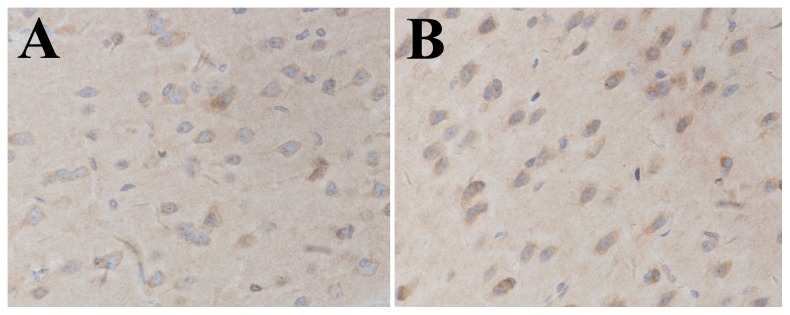
Localization of activated NF-κB detected by immunohistochemistry. NF-κB p65 immunoactivity was mainly presented in neurons in the SAH group. Furthermore, NF-κB p65 immunoactivity mainly located in the nuclei of neurons.

We examined the localization of activated NF-κB in the brain by immunohistochemical staining. It was shown that NF-κB p65 immunoactivity was mainly presented in neurons, especially in the nucleus, which implied that NF-κB was activated after SAH (Fig.2).

### Effect of PDTC on cell injury in the brain

**Figure 3:**
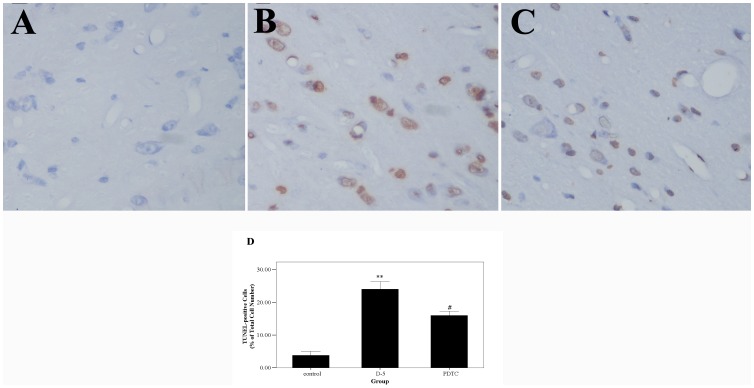
The cell injury in the brain was assessed by detecting the DNA fragmentation using TUNEL staining. Panel 1. It was shown that the cells have a normal structure and are light blue-stained. In the SAH group, the dystrophic and brown-stained TUNEL-positive cells are observed in the cortex. And the TUNEL-positive cells in the PDTC group are less than that in the SAH group. Panel 2. Quantification of the TUNEL staining showed that the TUNEL-positive cells are significantly increased in the SAH group compared with that in the control group. In the PDTC group, the TUNEL-positive cells are significantly decreased compared with that in the SAH group. **P<0.01 vs. control group, #P<0.05 vs. SAH group.

**Figure 4:**
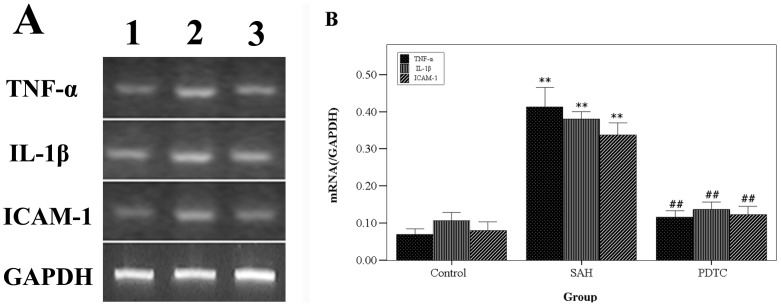
The gene expressions of TNF-α, IL-1β, and ICAM-1 in the brain. The representative autoradiograms of the RT-PCR results of TNF-α, IL-1β, ICAM-1. B) Relative amount of TNF-α, IL-1β and ICAM-1 mRNA. The levels of TNF-α, IL-1β and ICAM-1 mRNA increased after SAH and was suppressed in the PDTC group. **P<0.01 vs. control group, ##P<0.01 vs. SAH group.

Cell injury in the brain was assessed by detecting the DNA fragmentation using TUNEL staining (Fig. 3A, B, C, and D). The TUNEL-positive cells mainly distribute on the dorsal temporal cortex and the temporal poles. The number of TUNEL-positive cells was much higher in the day-5 SAH group (group 3) compared with the control group (P<0.01). We intracisternally administrated the inhibitor of NF-κB, PDTC, in the SAH model in group 5. It was found that NF-κB DNA-binding activity in the group 5 decreased significantly compared with group 3 (P<0.01), which indicates that intracisternal administration of PDTC could effectively inhibit the DNA-binding activity of NF-κB in the brain. The number of TUNEL-positive cells in the group 5 with PDTC treatment also decreased compared with the group 3 (P<0.05). In addition, the levels of the TNF-α, IL-1β, and ICAM-1 mRNA in the brain were detected by RT-PCR (Fig. 4A and B), which were all significantly increased in the SAH group (P<0.05 vs. control group). There are statistical differences between the levels of TNF-α, IL-1β, and ICAM-1 mRNA in the group 3 and the group 5 (P<0.05).

## Discussion

The main findings of the present study are: 1) NF-κB DNA-binding activity increased after SAH, and peaked on day 3 and 5, which could all be suppressed by PDTC, an inhibitor of NF-κB; 2) cell death in the brain after SAH could also be alleviated by intracisternal administration of PDTC; 3) NF-κB may contribute to delayed brain injury via regulating the gene expressions of TNF-α, IL-1β, and ICAM-1 in the brain.

### The time course of NF-κB activation in the brain after SAH

The up-regulation of NF-κB DNA-binding activity occurred early after SAH. NF-κB DNA-binding activity was increased and almost reached the peak on *day 3*, the first day after the second blood injection, which might result in the early brain injury after SAH. Many pathological factors can induce the activation of NF-κB in the brain or in the neurons in vitro, such as elevation of glutamate[23], focal or global cerebral ischemia[9], [24], [25], and free radical generation[26]. Those events also occur in the brain immediately after SAH, which may result from the raised intracranial pressure (ICP), reduced cerebral blood flow (CBF) and cerebral perfusion pressure (CPP), and dysfunction of cerebral auto-regulation[27], [28].

Nevertheless, the up-regulation of NF-κB DNA-binding activity on *day 5* and *day 7* might contribute to delayed brain injury. In another study, we demonstrated that the delayed cerebral vasospasm in this two-hemorrhage SAH model began on *day 5* [29]. Thus, the pathological alteration in the brain caused by cerebral vasospasm-induced brain ischemia may result in the molecular alteration in the brain, which might include the up-regulation of NF-κB DNA-binding activity since abundant data suggest the activation of NF-κB induced by cerebral ischemia [30], [31], [32], [33]. On *day 7*, NF-κB DNA-binding activity decreased profoundly compared with that on *day 3* and *day 5*, which accords with the recovery of cerebral vasospasm in this two-hemorrhage SAH model (in this animal model, the time course of cerebral vasospasm after SAH was similar but not the same as that in humans)[29]. These findings implied that cerebral vasospasm could induce the pathological alteration in the brain including activation of NF-κB. Further studies were encouraged to clarity the details between the delayed cerebral vasospasm and the activation of NF-κB in the brain after SAH.

Moreover, NF-κB was demonstrated to be activated in the brain and to play a crucial role in brain injury after cerebral ischemia or traumatic brain injury via regulating the expressions of inflammatory genes [6], [7], [9]. Thus, the NF-κB pathway is very critical for the pathophysiology after brain injury. In the present study, the gene expressions of the cytokines and adhesion molecules were also up-regulated in the brain after SAH, which is consistent with one previous study from other group[2]. Because of the key role of the activated NF-κB in inflammation, it would be reasonable to assume that inhibiting the NF-κB can ameliorate the inflammation in the brain post injury and then attenuate the entire damage process after SAH. Therefore, in this study, we used the PDTC, a confirmed effective inhibitor of NF-κB. It was demonstrated to inhibit the NF-κB DNA-binding activity in the brain and to reduce the levels of TNF-α, IL-1β, and ICAM-1 gene expressions.

Taken together, all of these findings indicated that NF-κB plays a potential role in pathophysiology of the brain after SAH in rabbits. Its long-lasting action pattern makes it to be a possible target for pharmacological intervention in brain injury after SAH.

### NF-κB and delayed brain injury

Some studies have reported that DNA fragmentation or cell death occurred in the brain in different SAH models[34], [35], [36], [37]. But the underlying mechanisms have not been fully identified yet. Also, it has been reported that heat shock proteins were induced by experimental SAH *in vivo* or by the blood products in the subarachnoid space *in vitro*. They were regarded as the sensitive markers of neuronal injury and were believed to be related to the cell death after SAH[35], [37], [38]. Additionally, Prunell and his colleagues' study indicated that inflammatory reaction in the brain might have significant contributions to neuronal injury after SAH[2]. In the present study, the gene expressions of the cytokines and adhesion molecular showed a strong correspondence with the degree of DNA fragmentation.

Although NF-κB plays a key role in regulating the inflammatory reaction, the role of NF-κB in cell death and cell survival after brain injury remains controversial. It appears that NF-κB is involved in both processes, not only *in vitro* experiments but also *in vivo* studies[39], [40], [41], [42], [43], [44], [45], [46], [47], [48], [49]. Thus, we speculated that a balance could be present in the role of NF-κB in inducing and reducing cell death in the brain. Recently, several studies showed that NF-κB contributed to the cell death in cerebral ischemia [25], [39], [43], [50]. Our results clearly showed that inhibiting the DNA binding activity of NF-κB could attenuate the cell death in the brain after experimental SAH.

The mechanism by which NF-κB contributes to cell death might be the genes regulated by NF-κB. NF-κB activation can lead to the increase of gene expression of some factors such as TNF-α, IL-1β, ICAM-1 and nitric oxide synthetase (NOS)[46], [50], which are all believed to aggravate the cell death in the brain. However, the exact mechanisms need to be clarified in further studies. Characterization of the relationship between NF-κB activation and DNA fragmentation or cell death in the brain after SAH may result in the identification of target molecules to develop new therapeutic interventions.

### PDTC and delayed brain injury

PDTC has been characterized as a relatively selective inhibitor of NF-κB activation mainly by preventing the degradation of I-κB[19], [50]. In addition, PDTC has metal-chelating and antioxidant properties, both of which can mediate NF-κB inhibition [19], [50]. PDTC has been reported to stimulate cell death by suppressing activation of NF-κB in various cancer cells[49], [51]. And some previous studies also showed the neuroprotective effects of PDTC in brain injury, which was also mediated by suppressing activation of NF-κB[19], [50]. In the present study, PDTC was demonstrated to suppress the activation of NF-κB and to ameliorate the cell death in the brain after SAH. Hence, it is reasonable to draw a conclusion that the neuroprotective effects of PDTC were also mediated by suppressing the activation of NF-κB.

A previous study has reported that PDTC analogous could inhibit HIV progression in patients, which suggests the broad prospects for the clinical application of PDTC[52]. PDTC and its structurally modified derivatives with possible greater efficacy may represent potential therapies against SAH.

### Limitations

Some limitations of the present study should be noted. First, PDTC has the antioxidant capacity, and was regarded as an antioxidant agent in many studies [53], [54]. Therefore, it is possible that the beneficial effect of PDTC is at least partially based on its antioxidative functions. Second, although we used this two-hemorrhage rabbit model of SAH aiming to produce severe vasospasm and brain injury similar to that in humans[22], the effect of vasospasm on the delayed brain injury should be examined in this model. In addition, another study of our group has showed that cerebral vasospasm could be attenuated partially by PDTC in this animal model[29], so studies exploring the detailed mechanisms of delayed brain injury have been initiated in our lab. Third, because cell death caused by early brain injury was failed to be excluded from that detected on day 5 post SAH in this study, it is essential to validate our findings in dog SAH models, in which the interval between early brain injury and delayed brain injury would be longer.

### Conclusion

Activated NF-κB in the neurons after SAH contributes to delayed brain injury by regulation of the gene expressions of the cytokines and ICAM-1 in the brain. The NF-κB inhibitor, PDTC, could attenuate the delayed brain injury after SAH.
